# TLR3 activation of microglia-containing cerebral organoid induces antiviral factors against HIV-1 infection

**DOI:** 10.3389/fphar.2026.1835457

**Published:** 2026-06-30

**Authors:** Qian-Hao Xiao, Xu Wang, Priyanka Sarkar, Xiao-Long Wang, Li Song, Binhua Ling, Wen-Hui Hu, Wen-Zhe Ho

**Affiliations:** 1 Department of Pathology and Laboratory Medicine, Philadelphia, PA, United States; 2 Center for Substance Abuse Research, Temple University Lewis Katz School of Medicine, Philadelphia, PA, United States; 3 Host-Pathogen Interactions Program, Texas Biomedical Research Institute, San Antonio, TX, United States; 4 Department of Neuroscience and Anatomy, Virginia Commonwealth University, Richmond, VA, United States

**Keywords:** HIV, IFNs and ISGs, iPSC-derived microglia containing cerebral organoids, Poly I:C, TLR3

## Abstract

Human induced pluripotent stem cell (iPSC)-derived cerebral organoids have been increasingly used as a brain model for studying various neurological disorders and neurotropic virus infections, including HIV-1. However, it is unclear whether iPSC-derived cerebral organoids possess functional innate antiviral immunity against HIV-1. In this study, we examined Toll-Like Receptor 3 (TLR3) activation and its role in the induction of IFNs and interferon-stimulated genes (ISGs) against HIV-1 in human iPSC-derived microglia containing cerebral organoids (MCOs). We observed that MCOs possess functional TLR3, which could be effectively activated by poly I:C. TLR3 activation of MCOs resulted in HIV-1 inhibition and induction of IFN-β/IFN-λ, antiviral ISGs (MX1, MX2, GBP5, SAMHD1, Viperin, and ISG56), and CC chemokines (MIP-1α, MIP-1β, and RANTES), the ligands for HIV entry coreceptor CCR5. This TLR3 activation-mediated anti-HIV-1 effects could be blocked by a specific TLR3 inhibitor. These findings indicate that human iPSC-derived MCOs are a suitable model for investigation of brain immunity and HIV-1 infection.

## 1 Introduction

Toll-like receptor 3 (TLR3) in conjunction with TLR7 and TLR9 constitutes an efficient sensor system to detect viral infections. While TLR7 and TLR9 can be triggered by single-stranded RNA viruses and cytosine phosphate guanine DNA, respectively (Cen et al., 2013; Guo et al., 2018; Meng et al., 2021; Xu et al., 2019), TLR3 recognizes double-stranded RNA viruses (Tissari et al., 2005) and triggers antiviral responses. TLR3 activation can be mimicked in experimental settings using synthetic analogs such as poly I:C. Importantly, TLR3 activation by its ligands triggers IFN signaling pathway and induces the production of both type I and type III IFNs as well as interferon-stimulated genes (ISGs). Although HIV is a single-stranded RNA virus that can be detected by TLR7 (Meng et al., 2021), it can be recognized by TLR3 as well (Brulé et al., 2002; Zhou et al., 2010) because HIV forms double-stranded RNAs during its replication. In HIV-1 infection of the brain, viral RNA released from either outside the blood brain barrier or HIV-1-infected cells can activate the TLR-IFN signaling pathway in microglia, triggering the antiviral response in the brain. A study showed that primary human fetal microglial cells possess TLR3 which could be activated and induce IFN regulatory factor 3 (IRF3)-dependent anti-HIV effect (Suh et al., 2009). We reported that TLR3 activation potently inhibited HIV infection of primary human macrophages through producing the multiple intracellular viral restriction factors (Zhou et al., 2010; Sang et al., 2014; Dai et al., 2015). In addition, TLR3 signaling activation of human iPSC-derived microglia could induce antiviral cellular factors against HIV-1 (Wang et al., 2023; Altahrawi et al., 2025). While these observations with macrophages and microglia cultures are significant, further studies using a human brain model are necessary to verify the role of TLR3 in the brain innate immunity against HIV-1.

The brain innate immunity depends primarily on the functions of glial cells, especially microglia which are important for the early control of viral replication and clearance. Microglial cells produce antiviral factors against viral infections, including HIV-1. Therefore, the brain may rely heavily on innate immune response to prevent and control HIV-1 infection and establishment of HIV-1 latency (Geffin and McCarthy, 2013). In the brain microenvironment, microglial functions significantly depend on their direct and/or indirect contact with other brain cell types such as neurons and astrocytes. Therefore, it is clinically relevant and significant to develop a 3D brain organoid model with all major brain cell types. Recently, human induced pluripotent stem cell (iPSC)-derived cerebral organoids have been increasingly used as a brain model for studying various neurological disorders and neurotropic virus infections. Several groups have demonstrated that the human brain organoids with microglia are susceptible to HIV-1 infection (Dos Reis et al., 2022; Gumbs et al., 2022; Donadoni et al., 2024; Narasipura et al., 2025; Wei et al., 2023; Kong et al., 2024). However, it is unclear whether these brain organoids possess functional innate antiviral immunity against HIV-1 infection. In this study, we examined whether human iPSC-derived microglia containing cerebral organoids (MCOs) express functional TLR3 which could be activated by poly I:C and IFNs and interferon-stimulated genes (ISGs). In addition, we examined the mechanisms for TLR3 activation-driven HIV-1 inhibition in MCOs.

## 2 Materials and methods

### 2.1 Human pluripotent stem cell cultivation

Experiments were performed utilizing four different human iPSC lines: WT10, WT11, WT15, which were obtained from Human Pluripotent Stem Cell Core at the Children’s Hospital of Philadelphia (CHOP) (Children's Hospital of Philadelphia, 2024), and the iPSC line IPS11 was generated from ALSTEM (Cat# iPS11, ALSTEMBIO). Prior to organoid generation, iPSCs were expanded in feeder-free monolayer culture on Matrigel coated plates (STEMCELL technology). All the iPSC lines were cultured in a 6-well plate as monolayer on 0.1 mg/mL Matrigel (Corning) in DMEM/F12 (Gibco) media. The cells were maintained in mTeSR™ Plus media (STEMCELL Technologies) and kept at 37 °C with 5% CO_2_ with fresh media change every other day. Once 60%–70% confluency was reached, the cells were split with mechanical dissociation by using 0.5 mM ultrapure EDTA (Invitrogen) in PBS.

### 2.2 Human iPSC-derived microglia containing cerebral organoids (MCOs)

**FIGURE 1:**
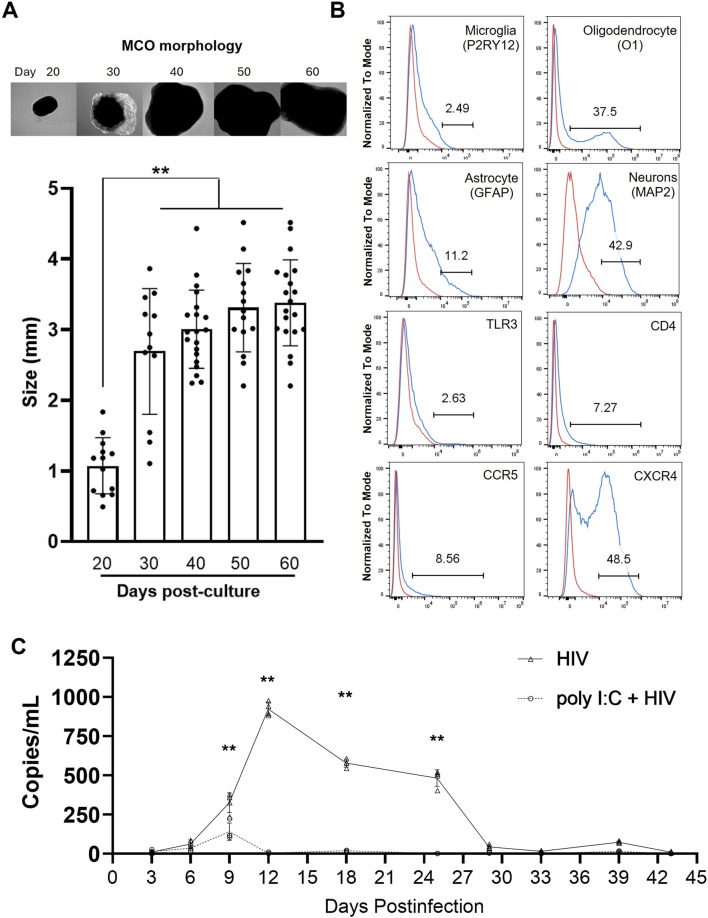
Characterization and effect of poly I:C on HIV infection of microglia-containing organoids (MCOs). **(A)** MCOs morphology from a representative iPSC line donor (upper panel) and size distribution (lower panel) during 60-day culture. Each dot represents a single organoid, and size (mm) was measured using ImageJ. Data are presented as mean ± SD relative to organoids at the age of Day 20. Asterisks indicate significant differences between groups (**P < 0.01). **(B)** Flow cytometry analysis of cell markers, HIV entry receptors and TLR3 in MCOs. As indicated: P2RY12 (microglial marker), O1 (oligodendroglial-lineage marker), GFAP (astrocytic-lineage marker), and MAP2 (neuronal marker). Marker expression reflects lineage association rather than terminal maturation. In addition, HIV entry receptors (CD4, CCR5, CXCR4) and TLR3 positive cells were examined. Percentages of positive cells were calculated from viable singlet cells. **(C)** 50 day-cultured MCOs were pretreated for 12h with the TLR3 agonist Poly (I)C (10 μg/mL), then infected with live HIV-1Bal (2 ng p24 per well in 48-well plate) for 48h. Cultures were maintained with poly I:C, and viral replication was monitored by measuring HIV Gag RNA in the supernatant over the indicated infection period. Data are presented as mean ± SD from organoid cultures derived from four independent iPSC lines, with triplicate cultures per condition, across two independent experiments.

Human iPSC-derived cerebral organoids were generated with the STEMdiff™ Cerebral Organoid Kit (STEMCELL technology) as depicted in Figure 1A. On day 0, iPSC colonies were dissociated into single cells with Versene (Gibco). A total of 9000 cells/well were plated in ultra-low attachment 96-well plates (Corning Costar) and maintained in 100 µL of EB Seeding Medium (STEMdiff™ Cerebral Organoid Basal Medium 1, supplemented with STEMdiff™ Cerebral Organoid Supplement A, and 10 µM Y-27632). At day 2 and 4, 100 µL of EB Formation medium (STEMdiff™ Cerebral Organoid Basal Medium 1 supplemented with STEMdiff™ Cerebral Organoid Supplement A) was added to the culture. At day 5, embryoid bodies were transferred to a 24-well ultra-low attachment plate (Corning Costar) and cultured in Induction Medium (STEMdiff™ Cerebral Organoid Basal Medium 1 and STEMdiff™ Cerebral Organoid Supplement B). At day 7, embryoid bodies were transferred into cold Matrigel (hESC-qualified Matrix, Growth Factor Reduced, BD Matrigel™, Corning) droplets and subsequently incubated at 37 °C with 5% CO_2_ for 30 min to induce Matrigel polymerization. Then, the Matrigel droplets embedding the embryoid bodies were incubated in Expansion Medium (STEMdiff™ Cerebral Organoid Basal Medium 2 with STEMdiff™ Cerebral Organoid Supplement C and STEMdiff™ Cerebral Organoid Supplement D) in a 6-well ultra-low attachment plate (Corning Costar). At day 10, organoids were placed in maturation medium (STEMdiff™ Cerebral Organoid Basal Medium 2 and STEMdiff™ Cerebral Organoid Supplement E in a 49:1 ratio) in an incubator with orbital shaker (65 rpm), at 37 °C with 5% CO_2_, and kept in these conditions until the end of the experiment.

### 2.3 MCOs morphology

Brightfield micrographs were captured using an EVOS imaging system (Thermo Fisher Scientific) and subsequently analyzed using ImageJ (National Institutes of Health, Bethesda, MD, United States of America). Diameter of organoid was measured every 10 days. The size was calculated as the average of the major axis and its orthogonal. Organoids were excluded from measurements if: 1) the center of the vesicle-like object was opaque, 2) debris obscured ability to define inner and outer epithelial boundaries, and/or 3) the structure was incomplete.

### 2.4 PolyI:C and TLR3 inhibitor treatment

The experiments were conducted with MCOs at age of 50–60 days. To activate TLR3, polyinosinic-polycytidylic acid (poly I:C, HMW, Invivogen) was mixed with LyoVec (Invitrogen, United States of America) and the mixture was added into MCOs cultures. To inhibit TLR3 activation, MCOs were incubated with the TLR3/dsRNA complex inhibitor (TCI, cat#: 614310, Merck) for 4h prior to addition of poly I:C. At the indicated time points, MCOs were harvested for RNA extraction or protein analysis.

### 2.5 Cell viability assay

Single cells dissociated from MCOs were plated in 96-well plates (2 × 10^4^ cells/well) and treated with poly I:C or TLR3/dsRNA complex inhibitor (TCI) for 48 h at 37 °C. CellTiter 96® Aqueous One Solution Cell Proliferation Assay Kit 8 (Promega) was used to measure the cell viability.

### 2.6 Quantitative PCR

Total RNA from MCOs was extracted using AllPrep DNA/RNA/Protein Mini Kit according to handbook. Quantification of RNA sample concentrations and purity was measured using the NanoDrop spectrophotometer (Thermo Fisher Scientific). For each sample, total RNA (1 µg) was subjected to reverse transcription (RT) using reagents from Promega (Promega, WI, United States of America). The RT reaction was performed with random primers for 1h at 37 °C. The reaction was terminated by incubating the reaction mixture at 99 °C for 5 min, and the mixture was then kept at 4 °C. The resulting cDNA was then used in triplicate reactions of quantitative PCR reactions (RT-PCR). The RT-PCR was performed with iQ SYBR Green Supermix (Bio-Rad Laboratories, CA, United States of America). Thermal cycling conditions were designed as follows: initial denaturation at 95 °C for 3 min, followed by 45 cycles of 95 °C for 10 s, and 60 °C for 1 min. PCR cycle number at threshold is represented as Ct. Relative expression levels of genes of interest were quantified by 2^−ΔΔCT^ method after normalization for GAPDH expressed in fold change as compared to corresponding uninfected control cells.

### 2.7 RT^2^ profiler PCR array

Total RNA was analyzed using the human type I IFN response RT^2^ Profiler PCR array (cat#: PAHS-016Z; QIAGEN), which profiles the expression of 84 gene transcripts that are known to be involved in the type I IFN response, as well as the expression of five housekeeping genes (ACTB, B2M, GAPDH, HPRT1, and RPLP0). In addition, one well contains a genomic DNA control, three wells contain reverse transcription controls, and three wells contain a positive PCR control. For each sample, 500 ng of RNA was reverse transcribed into cDNA using the RT^2^ first-strand kit (QIAGEN, United Kingdom). The cDNA was then mixed with the RT^2^ SYBR Green Master mix (QIAGEN, United Kingdom) and nuclease-free water. Next, 25 µL of the PCR mix was added to each well of the 96-well plate. All steps were done according to the manufacturer’s instructions. The RT-PCR reaction was run on a QuantStudio™ 3 Real-Time PCR System according to the following conditions: 95 °C for 10 min, followed by 45 cycles of 95 °C for 15 s and 60 °C for 1 min. Data analysis was conducted using a software-based tool (Applied Biosystems). Expression levels were quantified relative to the values obtained for housekeeping genes.

### 2.8 Flow cytometry

MCOs were washed 3 times in ice-cold DMEM/F12 and then dissociated by Papain. Briefly, MCOs were dissociated in a 1 mL Papain for 45 min mechanically disrupted every 15 min. The cell suspension was then reverse filtered through a 100 μm strainer (Thermo Fisher) and collected in a 15 mL falcon tube. The Douncer was washed twice with 1 mL DMEM/F12, added to the tube and the cells were centrifuged at 300 *g*, at 4 °C for 5 min. Afterwards, cell counting and viability test was performed, and cells were resuspended in ice-cold FACs buffer (PBS supplemented with 2% BSA and 0.5 mM EDTA). The cells were first blocked for 10 min with Fc Blocker (cat#: BDB564765, BD Biosciences) and subsequently stained with antibodies (CD4-Pacific Blue cat#: 300521, Thermofisher; CCR5-APC cat#: 561748, BD Biosciences; CXCR4-PE/Cy7 cat#: 12G5, BioLegend; P2RY12-PE cat#: S16001E, BioLegend; GFAP-Alexa488 cat#: GA5, Thermofisher; MAP2-488 cat#: CL488-57015, Proteintech) or an isotype-matched antibody to determine the background fluorescence. After 30 min at 4 °C, the samples were acquired on Cytek Aurora (Cytek Biosciences), a sequential gating strategy was applied to ensure data quality and accurate population identification. Briefly, debris was excluded based on forward and side scatter properties, followed by singlet discrimination using FSC-A versus FSC-H. Live cells were then gated using a eBioscience™ Fixable Viability Dye eFluor to exclude dead cells. Marker-positive populations were subsequently identified within the live singlet gate and the data analyzed using FlowJo v10.10 (Tree Star).

### 2.9 Phagocytosis assay

Primary human peripheral blood monocyte-derived macrophages (MDMs) and human iPSC-derived MCOs were incubated with pHrodo Red Zymosan BioParticles (5 μg/mL, cat #: P35364, Thermofisher) at 37 °C for 4 h or 24 h, respectively. MCOs were then washed 2 times with warm medium to remove the unbound particles and re-suspended in colorless live image buffer. The uptake of particle conjugates was visualized using the Eclipse Ti2 microscope with a filter cube (Ex470/30, Em530/50) (Nikon camera).

### 2.10 ELISA

MCOs were plated in a 48-well plate (2 per well) and transfected with poly I:C. Culture supernatants were then collected and analyzed for IFNs and CC chemokines using a ELISA kits (DuoSet, R&D system). Briefly, 100 μL of standards or samples from each well were incubated for 2 h with detecting antibody prior to incubation with HRP conjugated 2^nd^ antibodies for 20 min. The plates were read for the absorbance at 450 nm with a wavelength correction at 540 nm was measured with a SpectraMax 340 plate reader (Molecular Devices, San Jose, CA, United States). The values were compared against a standard curve that was generated using known concentrations to calculate concentration in the samples.

### 2.11 Statistical analysis

GraphPad Prism 9.1 software (GraphPad Software, United States) was used to analyze data that were represented as means ± SD. The results were treated using two-way ANOVA, followed by multiple comparison tests for individual comparison to determine statistical significance.

## 3 Results and discussion

To assess the structural developmental trajectory of the brain organoids, we monitored the sequential stages of brain organoid maturation, progressing from initial spherical structures to increasingly complex and dense formations (Figure 1A). Following embedding in Matrigel on day 30, brain organoids exhibited key developmental features, including gross morphological features consistent with early neural tissue organization. By day 40, the cerebral organoids attained a diameter ranging from 3 to 5 mm, achieving macroscopic visibility. These morphological changes indicate successful differentiation and self-organization of neural progenitors, a hallmark of functional brain organoid development. MCOs’ size started stabilizing by day 50, with no further growth by day 60. These morphological changes are consistent with cerebral organoid maturation, and brightfield imaging reflects global structural features. Flow cytometry analysis identified populations expressing markers associated with major brain cell lineages, including oligodendroglial-lineage (O1^+^), astrocytic-lineage (GFAP^+^), neuronal (MAP2^+^), and microglial (P2RY12^+^) cells (Figure 1B). The marker expression alone does not imply functional maturity. Although these marker-positive populations likely represent early lineage-committed or precursor-like cells, they are consistent with the developmental stage of the organoids. In particular, microglial identity was operationally defined based on P2RY12 expression, acknowledging potential heterogeneity related to activation state. We recognize that no single marker can definitively establish microglial identity, particularly in cerebral organoid systems where developmental trajectories and cellular states may differ from those observed in the human brain. Nevertheless, P2RY12 has been widely used as a microglia-associated marker in both primary tissue and organoid studies and remains one of the most commonly applied markers for identifying homeostatic microglia-like populations in human brain organoids (Ormel et al., 2018; Xu et al., 2021). Accordingly, we interpret the detected P2RY12-positive population conservatively as microglia-like or microglia-associated cells rather than definitively assigning microglial identity. To assess innate immune–related functional properties within MCOs, we examined particle uptake using pHrodo Red Zymosan particles (Supplementary Figure S1). Fluorescent signal was detected within intact organoids, indicating organoid-associated particle uptake activity. Particle uptake observed in MCOs is interpreted conservatively as organoid-associated phagocytic activity, without attributing this function to a specific cell type.

**SCHEME 1:**
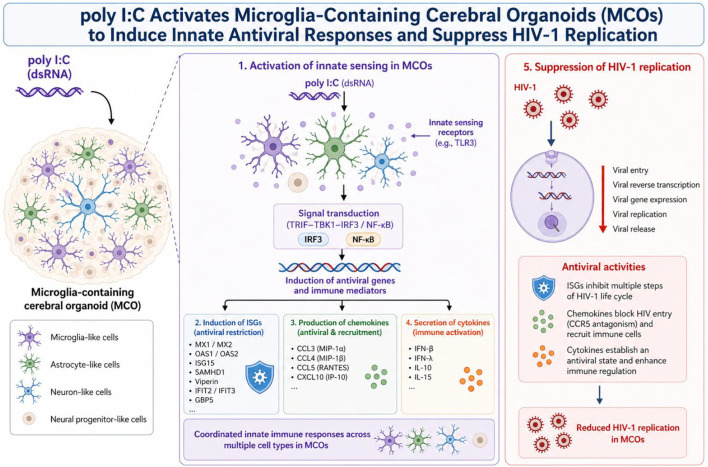
Poly I:C induces innate antiviral responses in MCOs to suppress HIV-1 replication. Poly I:C is recognized by intracellular pattern recognition receptors, triggering signal transduction pathways (e.g., TRIF–TBK1–IRF3 and NF-κB), which lead to the induction of antiviral genes and immune mediators. This activation results in: (1) upregulation of interferon-stimulated genes (ISGs) that establish intrinsic antiviral restriction; (2) production of chemokines that both exert antiviral effects and recruit immune cells; and (3) secretion of cytokines that promote immune activation and reinforce an antiviral state. Together, these coordinated innate immune responses act across multiple stages of the HIV-1 life cycle, including viral entry, reverse transcription, gene expression, replication, and release, ultimately suppressing HIV-1 replication within MCOs. Chemokine-mediated CCR5 antagonism and ISG activity contribute to blocking viral entry and inhibiting downstream replication steps, while cytokines enhance overall immune regulation and antiviral defense.

This study was designed to evaluate functional antiviral capacity downstream of TLR3 activation, rather than to dissect canonical intracellular signaling intermediates. A schematic overview of the proposed TLR3-mediated antiviral response pathway is shown in Scheme 1. Well-established components of the TLR3 pathway, including TRIF, IRF3, and NF-κB, have been extensively characterized in prior systems. Here, we focus on integrated functional outputs, interferon production, ISG induction, and suppression of HIV-1 replication, within a multicellular brain-like context. We next examined whether MCOs are suitable for studying HIV-1 infection and innate immune responses. MCOs expressed all ten Toll-like receptors as well as HIV-1 entry receptors CD4, CCR5, and CXCR4 (Supplementary Figure S2; Figure 1B), supporting their relevance as a brain-like model for host–virus interaction studies. Consistent with this, MCOs were productively infected by the CCR5-tropic HIV-1 BaL strain. Notably, stimulation with the synthetic dsRNA agonist poly I:C resulted in potent suppression of HIV-1 replication (Figure 1C), suggesting that activation of dsRNA-sensing innate immune pathways can effectively restrict viral infection in this system. The observed decline in HIV-1 replication at later time points likely reflects activation of intrinsic antiviral responses within the organoid system. HIV infection dynamics in the CNS are highly context-dependent, and immune-mediated restriction represents a biologically relevant outcome. While this system does not fully recapitulate long-term viral persistence, it is well suited to model early infection and innate immune control mechanisms. An additional limitation is that changes in the abundance, survival, or phenotypic state of P2RY12-positive microglia-like cells were not directly assessed following HIV-1 infection. Therefore, although the induction of interferons, ISGs, and antiviral chemokines supports a role for innate immune activation in suppressing viral replication, alternative explanations cannot be formally excluded. In particular, changes in susceptible microglial subpopulations, infection-induced cellular depletion, or shifts in cellular composition within the organoid may also contribute to the observed viral kinetics. Future studies incorporating longitudinal cellular profiling will be necessary to distinguish these possibilities.

**FIGURE 2:**
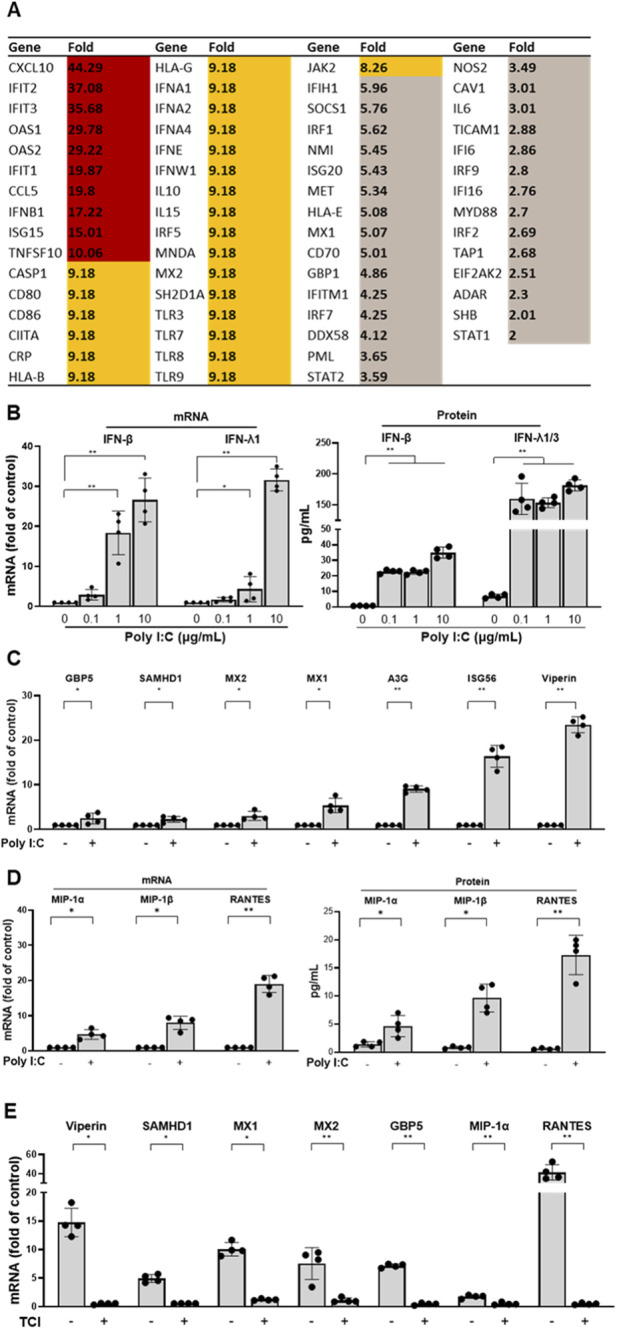
TLR3 activation of MCOs upregulates type I IFN pathway genes and induces CC chemokines. **(A)** Poly I:C treatment upregulates type I IFN pathway genes in MCOs. MCOs were treated with poly I:C (10 μg/mL) for 24 h and mRNA levels of 84 genes were assessed with a Type I IFN RT^2^ Profiler PCR array. The data shows gene expressions as absolute fold changes, with different colors indicating upregulation levels: brown (< 6-fold), orange (8–10 fold), and red (> 10-fold). Data are presented as mean ± SD of triplicate organoid cultures derived from a single iPSC line and are representative of findings consistently observed across four independent iPSC lines. **(B)** TLR3 activation of MCOs induces IFN-β and IFN-λ1. MCOs were treated with poly I:C at the indicated doses, and the supernatants or cell lysate were collected for RT-PCR and DuoSet ELISA at 24 h post-treatment. Data are presented as mean ± SD from organoid cultures derived from four independent iPSC lines, with triplicate cultures per condition, across two independent experiments (*P < 0.05, **P < 0.01). **(C)** TLR3 activation of MCOs induces ISGs. MCOs were treated with poly I:C (10 μg/mL), and the induction of the indicated ISGs was measured by RT-PCR at 24 h post-treatment. Data is presented as the fold change relative to the untreated MCOs. Data are presented as mean ± SD from organoid cultures derived from four independent iPSC lines, with triplicate cultures per condition, across two independent experiments (*P < 0.05, **P < 0.01). **(D)** TLR3 activation of MCOs induces CC chemokines. MCOs were treated with poly IC (10 μg/mL) and CC chemokines were analyzed by RT-PCR and ELISA at 24 h post-treatment. Data are presented as mean ± SD from organoid cultures derived from four independent iPSC lines, with triplicate cultures per condition, across two independent experiments. **(E)** TLR3 inhibitor blocks poly (I)C-mediated induction of the ISGs and CC chemokines. MCOs were pretreated with or without TLR3/dsRNA complex inhibitor (TCI, 12.5 µM) for 4 h prior to addition of poly I:C (10 μg/mL) to the cultures. The mRNA expression of the antiviral ISGs and chemokines were analyzed by RT-PCR. Data are presented as mean ± SD from organoid cultures derived from four independent iPSC lines, with triplicate cultures per condition, across two independent experiments (*P < 0.05, **P < 0.01).

Our earlier studies using human iPSC-derived microglia showed that TLR3 activation could induce the intracellular antiviral state characterized by the upregulation of IFNs, antiviral ISGs, and the CC chemokines which can restrict HIV-1 replication (Zhou et al., 2010; Wang et al., 2023). To characterize the molecular basis of this antiviral effect, we performed transcriptome analysis comparing control and poly I:C-treated MCOs. Poly I:C stimulation resulted in broad upregulation of interferon signaling pathways, with 62 of 84 interferon-related genes showing greater than twofold induction (Figure 2A). Upregulated genes included type I and type III interferons, interferon-stimulated genes, chemokines, and innate immune receptors. Increased expression of CD80 and CD86 was observed, consistent with enhanced innate immune activation rather than specific adaptive immune priming in the organoid context. Consistent with the transcriptomic data, poly I:C treatment induced dose-dependent increases in IFN-β and IFN-λ at both mRNA and protein levels (Figure 2B). The preferential induction of these interferons likely reflects integrated responses across multiple cell types within the organoid. Poly I:C stimulation also increased expression of antiviral ISGs, including MX1, MX2, GBP5, OAS1, ISG15, SAMHD1, and Viperin (Figure 2C), as well as CCR5-binding chemokines MIP-1α, MIP-1β, and RANTES (Figure 2D), all of which are known to contribute to restriction of HIV-1 replication. To assess the specificity of this response, MCOs were treated with a TLR3/dsRNA complex inhibitor prior to poly I:C stimulation. Inhibition of TLR3 signaling effectively blocked induction of interferons, ISGs, and chemokines (Figure 2E), supporting a TLR3-dependent mechanism underlying the observed antiviral phenotype. While this pharmacological approach supports pathway specificity, we note that contributions from additional nucleic acid–sensing receptors cannot be excluded. Given that microglia in this system arise through spontaneous mesodermal differentiation reported in unguided organoid cultures, and represent a relatively small fraction of total cells, we do not attribute antiviral effects exclusively to microglia. Rather, these responses likely reflect coordinated innate immune activation across multiple TLR3-expressing cell types within the organoid.

In summary, the present study provides experimental evidence that MCOs possess functional TLR3, stimulation of which by poly I:C activates IFN signaling pathway and induces the production of multiple antiviral cellular factors against HIV-1. Although poly I:C does not replicate all features of endogenous viral nucleic acids generated during HIV-1 infection, its strength lies in providing a robust, reproducible, and mechanistically well-defined stimulus to probe innate immune competence in a complex 3D brain-like system. A key consideration of this study is the use of poly I:C as a defined dsRNA agonist to activate TLR3. While poly I:C does not fully recapitulate endogenous viral sensing during HIV-1 infection, it provides a robust and reproducible stimulus to probe innate immune competence in a complex 3D system. In this context, poly I:C serves as a functional tool to determine whether MCOs possess the intrinsic capacity to mount TLR3-driven antiviral responses. HIV-1 neuroinvasion is likely associated with heterogeneous activation of multiple innate immune sensors (e.g., TLR7/8, RIG-I/MDA5), and modeling these dynamics remains a challenge. Thus, our findings establish a baseline framework of antiviral responsiveness rather than a direct model of physiological HIV sensing. Importantly, HIV-1 neuroinvasion is likely associated with heterogeneous and context-dependent engagement of multiple innate immune sensors, and modeling these dynamics remains a major challenge for *in vitro* brain systems. By establishing that MCOs can generate coordinated interferon and ISG responses downstream of TLR3 activation, our study provides a necessary functional foundation upon which more physiologically nuanced models can be built. These findings establish microglia-containing cerebral organoids as a functionally competent multicellular platform for studying innate antiviral responses in the human brain, while highlighting the need for future cell-type–resolved analyses to define the specific contributions of individual cellular populations.

## Data Availability

The original contributions presented in the study are included in the article/Supplementary Material, further inquiries can be directed to the corresponding author.
