# Supplementary material for: TLR3 activation of microglia-containing cerebral organoid induces antiviral factors against HIV-1 infection
Source: Front Pharmacol. 2026 Jun 30;17:1835457. doi: 10.3389/fphar.2026.1835457 (PMC13365852; doi:10.3389/fphar.2026.1835457)
Supplement: Supplementary file 1 [file DataSheet1.docx]

Supplementary Material

# Supplementary Figures


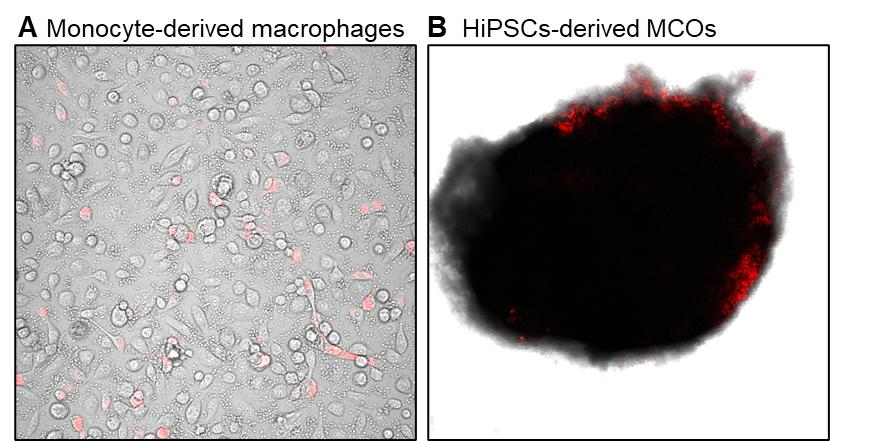


**Supplementary Figure 1.** MCOs showed phagocytic properties as macrophages. Phagocytosis capacity was analyzed in (A) primary human peripheral blood monocyte derived macrophages (MDMs) and (B) MCOs (day 50). MDMs and MCOs were incubated with 5 μg/mL pH-sensitive Zymosan for 4h and 24h, respectively, and imaged by a Nikon confocal microscopy.

**Supplementary Figure 1 Alternative Text.** Confocal microscopy images demonstrating the phagocytic capacity of MDMs and MCOs. **(A)** A bright-field confocal image overlaid with red fluorescence showing primary human monocyte-derived macrophages (MDMs). Multiple red, fluorescent puncta within the cell cytoplasm indicate the internalization of pH-sensitive Zymosan particles. **(B)** A confocal image of a day 50 HiPSC-derived MCO showing a dense, dark spherical mass with robust red fluorescent signaling concentrated along the outer periphery, indicating active phagocytosis of Zymosan particles by the integrated microglia.


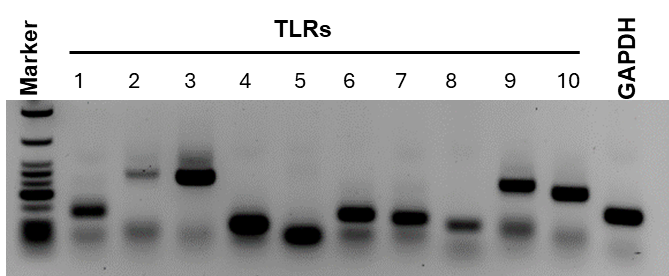


**Supplementary Figure 2.** Detection of TLRs (1-10) mRNA by RT-PCR in MCOs (day 50). cDNAs (amplified for 35 cycles) were separated on a 3% agarose gel containing ethidium bromide. 1-kb DNA ladder standard was used as a size marker.

**Supplementary Figure 2 Alternative Text.** RT-PCR analysis of Toll-like receptor (TLR) mRNA expression in MCOs at day 50. Image Description: An ethidium bromide-stained 3% agarose gel showing PCR amplification products for TLRs 1 through 10. A 1-kb DNA ladder is visible on the far left as a size marker. Distinct bands of varying molecular weights are present for all ten TLRs, confirming their expression in MCOs. GAPDH is shown on the far right as a positive internal loading control
